# Diagnostic Accuracy of Artificial Intelligence in Laryngeal Disorders: An Integrative Review

**DOI:** 10.3390/jpm16060301

**Published:** 2026-06-01

**Authors:** Samantha Mairesse, Antonino Maniaci, Giovanni Briganti, Jerome R. Lechien

**Affiliations:** 1 Department of Surgery, UMONS Research Institute for Language Science and Technology, University of Mons, 7000 Mons, Belgium; samantha.mairesse@umons.ac.be (S.M.); tnmaniaci29@gmail.com (A.M.); 2 Department of Medicine and Surgery, Enna Kore University, 94100 Enna, Italy; 3 Department of Neuropsychiatry and Computational Medicine, University of Mons, 7000 Mons, Belgium; giovanni.briganti@umons.ac.be; 4 Department of Psychiatry, HELORA Hospital, 7000 Mons, Belgium; 5 Department of Otolaryngology-Head and Neck Surgery, EpiCURA Hospital, 7331 Baudour, Belgium; 6 Department of Otolaryngology-Head and Neck Surgery, Foch Hospital, Paris Saclay University, 92150 Paris, France

**Keywords:** otorhinolaryngology, otolaryngology, artificial intelligence, laryngeal, disorders, disease, accuracy, machine learning

## Abstract

**Background/Objectives**: Laryngeal disorders are among the most prevalent conditions in otolaryngology, yet they remain challenging to diagnose without specialized expertise. Artificial intelligence (AI) systems leveraging machine learning (ML) and deep learning (DL) have demonstrated promising performance for the automatic detection and classification of voice disorders and laryngeal lesions. **Methods**: This review synthesizes findings from 88 studies published between 2015 and 2025 on AI-based laryngeal disorder detection, considering physioacoustic mechanisms, databases and acquisition protocols, AI architectures and validation strategies, and diagnostic performance. **Results**: The current literature supports high internal accuracies for binary healthy versus pathological detection (88–99%); meanwhile, performance decreases for higher-level tasks such as pathophysiological category classification and identification, particularly under external validation. From a clinical perspective, clinicians do not infer specific diagnoses from isolated acoustic parameters such as percent jitter or shimmer. Instead, they rely on how these perturbation patterns dynamically evolve during connected speech, where alterations guide perceptual differentiation between underlying disorders. Recurrent sources of bias include dependence on a limited number of historical vowel-based databases, class and demographic imbalance, and limited ecological validity of recording protocols. Additional concerns involve the predominant use of internal cross-validation and insufficient reproducibility or code sharing. **Conclusions**: Drawing on the literature, an integrative three-level clinical recognition framework is proposed, delineating realistic use cases for AI as a decision-support tool rather than an autonomous diagnostic system. Key priorities for future personalized medicine and research are also identified, including diversified multi-center datasets, standardized methodological reporting, rigorous external validation, and compliance with regulatory and ethical requirements for medical AI deployment.

## 1. Introduction

Nearly one-third of the adult population will experience a voice disorder during their lifetime [1], with dysphonia prevalence reaching 7% in the general population [2] and up to 50% among voice professionals [3], resulting in significant quality of life impairment and socioeconomic burden [4,5]. The clinical spectrum includes benign structural lesions (e.g., vocal fold nodules, polyps, and cysts [6,7]), neuromuscular disorders (e.g., vocal fold paralysis, spasmodic dysphonia [8,9]), inflammatory conditions, and malignant lesions [10,11]. While some conditions present with distinctive morphological patterns, most are associated with non-specific voice quality (VQ) modifications that remain difficult to attribute to a specific diagnosis through perceptual evaluation alone [10,12,13,14]. Indeed, perceptual VQ assessment is subjective, with significant inter- and intra-rater variability and disorder identification rates around 60–70% [15], further influenced by auditory fatigue, clinical experience, and cognitive biases [13]. Laryngeal imaging—including videolaryngoscopy, stroboscopy, and high-speed videoendoscopy—represents the reference standard for laryngeal evaluation [9,16,17,18]; however, access depends on practitioner expertise, equipment availability, and cost, while neuromuscular disorders may present without visible structural lesions, complicating diagnosis [19]. The European Consensus guidelines recommend multidimensional VQ assessment combining laryngostroboscopy with perceptual, patient-reported, and acoustic measures [5]. In this context, artificial intelligence (AI)-based acoustic analysis offers a non-invasive adjunctive approach for VQ diagnosis, triage, and follow-up [20,21,22,23,24]. Current AI architectures include classical machine learning (ML) models—such as support vector machines (SVMs), random forests (RFs), and Gaussian mixture models (GMMs) operating on hand-crafted acoustic features [6,7,25]—deep learning (DL) networks processing spectrograms or raw waveforms [3,26,27], and self-supervised encoders such as wav2vec 2.0 or HuBERT pre-trained on large unlabeled speech corpora [23,28,29]. Diagnostic performance ranging from 90% to 99% has been reported across acoustic and laryngeal image-based tasks [26,30,31,32,33,34].

This integrative review aimed to summarize AI system performance for laryngeal disorder detection and classification, while examining methodological biases limiting clinical translatability, including physioacoustic foundations, acquisition protocols, validation strategies, and regulatory and ethical implications.

## 2. Methods

The present work is a critical narrative review rather than a systematic review, reflecting the substantial heterogeneity of available studies in terms of populations, disorders, databases, VQ assessment methods, AI architectures and outcome measures. Although framed as a narrative synthesis, the literature search and study selection followed a structured approach inspired by systematic review methodology, with predefined eligibility criteria, multi-step screening and explicit documentation of recurrent biases. This approach allows the integration of converging findings, recurring methodological limitations, and identification of gaps requiring resolution before large-scale clinical deployment.

The structured bibliographic search was conducted in PubMed, Cochrane Library and Scopus by three independent investigators for the period 2015–2025. The search strategy combined MeSH terms—including *Dysphonia*, *Vocal Folds*, *Hoarseness*, *Laryngeal Neoplasms*, *Laryngopharyngeal Reflux*, *Voice Disorders*, *Phonation*, *Machine Learning*, *Deep Learning*, *Artificial Intelligence*, *Neural Networks*, and *Computer*—with non-MeSH keywords such as “laryngeal cancer,” “speech analysis,” “voice disorder,” “neural network,” and “classification.”

Eligible studies were original research papers applying AI methods—either classical ML approaches or DL architectures—to the recognition, detection, classification, or severity assessment of laryngeal disorders in human participants, with quantitative reporting of clinically interpretable diagnostic or severity-related outcomes. Studies were excluded if they (i) focused on non-laryngeal disorders, (ii) lacked explicit clinical application to vocal fold disorders, or (iii) consisted of purely technical works, case reports, or commentaries without empirical diagnostic evaluation. No restrictions were applied regarding patient age, language or specific laryngeal disorder subtype, provided that the study addressed a clinically relevant laryngeal diagnosis or severity outcome.

**Figure 1:**
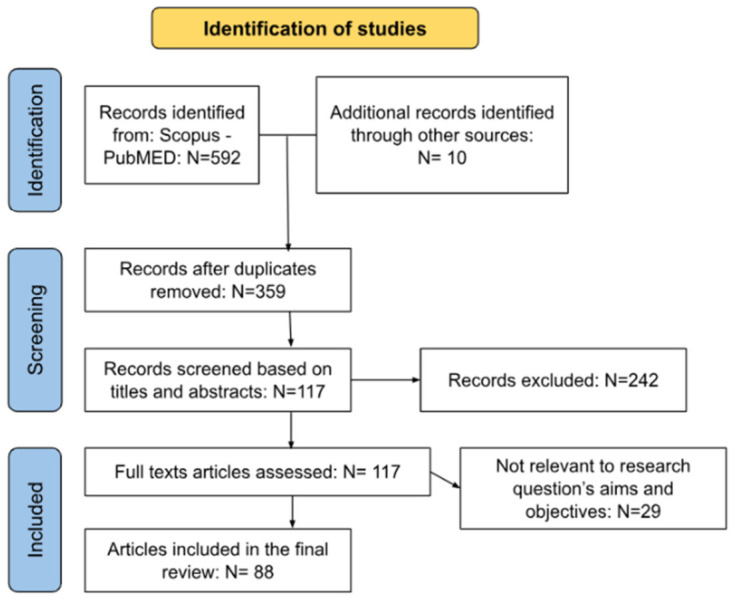
PRISMA flow chart.

Titles and abstracts were initially screened to exclude out-of-scope works, followed by full-text examination of potentially relevant papers to assess their contribution to at least one central theme of this review. For each included study, key variables were extracted in a structured manner, including population and disorder type, underlying database or recording protocol, AI architecture, input modality, validation strategy and main diagnostic performance metrics. Reference lists of included articles and relevant reviews were manually screened to identify additional studies missed by the electronic search. Given the substantial heterogeneity across included studies, methodological quality and potential biases were assessed qualitatively rather than through a standardized risk-of-bias tool. Recurrent limitations included reliance on restricted historical databases (Massachusetts Eye and Ear Infirmary, MEEI; Saarbrucken Voice Database, SVD), class imbalance, restriction to sustained vowel tasks, internal-only validation, and reporting limited to global accuracy metrics. Conversely, studies providing external validation, addressing noise robustness or device variability, and offering open-source documentation were considered to offer stronger and more generalizable evidence. The selection process is summarized in a PRISMA-inspired flow chart (Figure 1), detailing the number of records identified, screened, assessed for eligibility and ultimately included.

## 3. Results and Discussion

**Figure 2:**
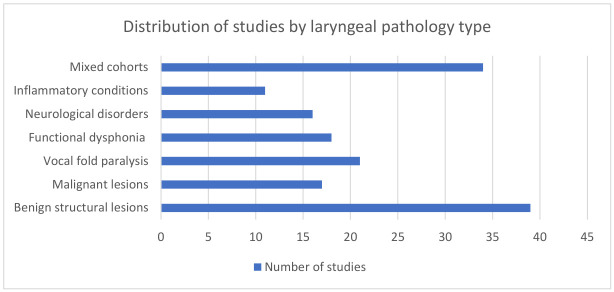
Distribution of included studies according to laryngeal pathology category.

A total of 88 papers were included. Benign structural lesions and vocal fold paralysis represent the most common disorders investigated with AI (Figure 2). Malignant lesions, neurological voice disorders and inflammatory conditions remain comparatively underrepresented. Based on the literature findings, authors have structured the review with the following key points: (i) physioacoustic foundation of automated detection of voice disorders; (ii) panorama of AI approaches; (iii) diagnostic performance and methodological biases; (iv) current clinical detection levels; (v) gaps and perspective; (vi) recommendations.

### 3.1. Physioacoustic Foundations of Automated Detection

#### 3.1.1. Source–Filter Model and Glottic Biomechanics

Automated detection rests on the assumption that structural or functional laryngeal alterations induce measurable acoustic modifications. The source–filter model remains the reference paradigm: voice results from the interaction between a glottic source driven by vocal fold oscillations and a supraglottic filter modulating spectral content [6]. Glottal inverse filtering and linear prediction residual signal analysis isolate the excitation component, providing a more physiologically relevant representation of vibratory irregularities induced by laryngeal disorders [6,35], with derived descriptors achieving classification performance exceeding 95% [35,36].

#### 3.1.2. Acoustic Correlates and Exploitable Features

Recent work systematically links acoustic signatures to perceptual profiles—roughness, breathiness and strain—demonstrating that a restricted set of spectral, cepstral and noise descriptors discriminate these deviations with accuracy exceeding 80% [37]. Laryngeal disorders generate exploitable signatures at distinct levels. Benign structural lesions modify vocal fold mass and vibratory symmetry, increasing cycle-to-cycle perturbations of fundamental frequency (F0) and amplitude while reducing harmonicity [4,6,7]. Unilateral paralysis generates persistent glottic insufficiency and air leakage [4,8], whereas essential vocal tremor produces characteristic periodic F0 and amplitude modulations between 4 and 8 Hz [9,31,38]. Convergence exists across studies regarding the acoustic parameters preferentially exploited by AI models. Leite et al. demonstrated that 15 of 34 extracted features—dominated by perturbation indices, harmonicity, cepstral descriptors and non-linear dynamics—were sufficient for 91% accuracy [39]. Li et al. showed that Mel frequency cepstral coefficient (MFCC)-derived statistics outperformed percent jitter and percent shimmer, achieving 96.4% accuracy in combination and 95.2% alone [40]. Naranjo et al. confirmed that a compact set mixing perturbation, cepstral peak prominence, glottal noise excitation and non-linear dynamics reached 89% cross-validated accuracy for Reinke’s oedema [41]. Nevertheless, these signatures remain physiologically non-specific, being shared across nodules, polyps, Reinke’s oedema, reflux-related changes and hyperfunctional dysphonia [39,40,41].

#### 3.1.3. Acoustic Overlap Between Pathophysiologically Distinct Conditions

Several pathophysiologically distinct disorders produce overlapping acoustic signatures, leading to inherent diagnostic ambiguity within the vocal signal itself [6,7,19,42]. This acoustic overlap explains why models achieve accuracies of up to 95% in binary healthy versus pathological classification tasks. In contrast, performance decreases to 70–85% when distinguishing between specific disorders, highlighting an intrinsic limitation that cannot be resolved through increased model complexity [6,7,24,25,42]. Spasmodic dysphonia, which is a neuromuscular disorder marked by dynamic laryngeal instability, and hyperfunctional dysphonia share nearly identical acoustic profiles, both featuring reduced harmonic-to-noise ratio (HNR) and F0 irregularity captured by jitter measures [4,9]. Vocal fold nodules and polyps generate comparable cycle-to-cycle perturbations in jitter and shimmer indices due to added mass and incomplete glottal closure, despite their distinct structural etiologies [4,6,23]. Unilateral vocal folds paralysis (UVFP) and spasmodic dysphonia also overlap acoustically, both yielding decreased harmonicity alongside increased glottic and subglottic noise [8]. This acoustic overlap reflects the fact that distinct laryngeal disorders may converge toward similar acoustic manifestations and therefore generate comparable measurable perturbations. As a result, this creates an intrinsic diagnostic ambiguity that cannot be resolved by deeper networks or additional model parameters alone. Multiple distinct pathophysiological mechanisms generate identical acoustically measurable perturbations, leading to mathematically inevitable diagnostic ambiguity. In clinical practice, this reality justifies why AI systems based on acoustic parameters measured on a sustained vowel cannot, currently, replace morphological information obtained from laryngeal videostroboscopy for definitive specific diagnosis [19]. Instead, AI serves best as a decision-support for binary triage, distinguishing healthy from pathological voices or for broad pathophysiological categorization, as will be developed further in Section 6. Note that, to date, AI has been rarely applied to connected text, which should make sense regarding the ability of experienced practitioners to recognize some vocal lesions based on the connected text of patients.

### 3.2. Panorama of AI Approaches

#### 3.2.1. Data Characteristics and Acquisition Protocols

##### Recording Modalities and Acoustic Conditions

Performance and robustness of automated detection systems depend decisively on data quality and experimental protocols. Most studies rely on recordings in acoustically controlled environments using quality microphones and standardized parameters [43,44], such as the European Consensus for Voice Quality [5]. Historical protocols frequently employ high sampling frequencies (25–50 kHz), although 16 kHz proves sufficient to capture relevant spectral components [45]. While optimal conditions favor reproducibility, they only partially reflect real clinical contexts where ambient noise, reverberation and recording device variability are frequent [46,47].

##### Vocal Tasks: Sustained Vowels or Connected Speech

Vocal task is another determining factor in reported performance, with most databases and consensuses recommending the acoustic analysis on sustained vowels, particularly the vowel /a/, produced at comfortable pitch and intensity [5,23]. This approach facilitates the extraction of ‘stable’ acoustic parameters and limits intra-subject variability. Indeed, approximately 82% of the reviewed studies rely exclusively on sustained vowels, reporting binary detection accuracies that are, on average, 5 to 10 percentage points higher than those obtained via connected speech tasks in similar architectures [8,48]. While sustained vowels remain the gold standard due to their signal stability, they fail to capture the dynamic laryngeal instability present in natural speech. Empirical evidence suggests that connected speech tasks provide superior diagnostic sensitivity, particularly for neuromuscular disorders and functional dysphonia, as they involve complex coordination and prosodic variations that are often masked during static phonation [8,9,19]. Consistently, clinical studies have shown that certain phenomena (e.g., diplophonia) are not systematically elicited by standardized vowels or short reading tasks. For instance, it has been observed that only 70% of clinically confirmed diplophonic segments are detectable during sustained phonation tasks [19,49]. This indicates that some disorders manifest intermittently and may be underrepresented in sustained-vowel protocols. Conversely, continuous/connected speech analysis enables the capturing of dynamic and intermittent vocal phenomena that are unlikely to appear during standardized vocal tasks and may provide more ecologically valid assessment [19,40,49]. From a clinical standpoint, experienced clinicians do not infer specific laryngeal diagnoses from isolated acoustic parameters such as percent jitter or shimmer measured on sustained vowels. Instead, they rely on how these perturbation patterns evolve dynamically during connected speech, where characteristic alterations guide perceptual differentiation between underlying disorders. Nevertheless, continuous speech may introduce an increased variability related to linguistic content, prosodic patterns, and articulatory demands, complicating extraction of directly comparable acoustic signatures between individuals [38,49].

##### Databases and Representativeness Bias

A limited number of databases have been used recurrently in the literature [4,6,7,38,48,50,51,52,53]. Despite their significant contribution to the development of automatic VQ detection methods, several limitations were reported across multiple studies. Both databases rely predominantly on sustained vowel recordings, include a restricted number of disorders compared to full clinical diversity, and are often imbalanced between normal and pathological classes [6,23,38]. Furthermore, historical databases have frequently been constituted under different recording conditions for healthy and pathological subjects, inducing potential acquisition bias. Learning algorithms may therefore exploit these recording differences instead of capturing actual pathophysiological characteristics [11,14,54]. Table 1 summarizes the five voice databases that were the most frequently used across the studies included in this review, highlighting their core characteristics and documented methodological biases.

##### Ecological Conditions and Noise Robustness

Robustness of automated detection systems to ambient noise represents a critical issue for clinical applicability, yet few studies explicitly examine noise impact under realistic conditions [46,50]. Recent research demonstrates that some approaches even maintain high performance under unfavorable signal-to-noise ratios [31,42]. Intelligent denoising approaches based on probabilistic noise models and perceptually motivated time–frequency representations have been proposed to improve signal quality exploitable by AI models. These approaches highlight the necessity of integrating real acoustic constraints from the system design phase, particularly when systems are intended for telemedicine use or uncontrolled clinical contexts [29,42,55]. Specialized architectures such as Long Short-Term Memory (LSTM) networks optimized by multi-objective denoising criteria have been shown to significantly improve speech signal-to-noise ratio while preserving vocal structure relevant for automated pathology detection [31,50,55].

#### 3.2.2. Classical ML and Feature Engineering

Classical ML methods operate on explicitly defined acoustic feature vectors and have demonstrated high performance, particularly when databases are of limited size and vocal tasks are standardized [6,7,25]. Their success is partly explained by a close alignment with physiological foundations of pathological phonation. Descriptors, such as temporal measures, frequency-based indices and reconstructed glottic-signal features can be directly linked to specific biomechanical mechanisms, like the vibratory irregularity, glottic turbulence or closure asymmetries [6,36]. This mapping between acoustic descriptors and underlying physiology confers appreciable interpretability to classical ML models used for automatic vocal fold disorder detection [36,39,40].

#### 3.2.3. Deep Architectures and Representation Learning

Compared with classical feature-based models, DL architectures enable partial emancipation from manual feature engineering by automatically learning hierarchical representations from raw or weakly transformed data [24,26,40]. This advantage becomes particularly evident as the number of disorders increases and intersubject variability remains substantial [26]. Convolutional neural networks (CNNs) have been widely applied to vocal spectrograms and laryngoscopic images [24,33], while recurrent and bidirectional architectures capture temporal dynamics of pathological phonation [16,56,57]. Pre-trained self-supervised models—wav2vec 2.0 and HuBERT (Hidden-Unit BERT) —are used as generic acoustic feature extractors, yielding significant performance gains over hand-crafted descriptors when annotated data are limited [23,58]. Similar benefits arise from biologically inspired transforms, including scalograms and gammatonegrams [29,59,60]. Comparative evidence suggests that architectural families are better suited to specific data regimes than any universally optimal model [16,26,56,61]. On small datasets, classical ML classifiers achieve 90–96% accuracy while remaining computationally lightweight and interpretable [39,40]. Specifically, studies using SVMs on limited cohorts report an area under the curve above 0.92 for polyp detection [40]. Lee and Lee demonstrated on the SVD that SVMs trained on high-dimensional ComParE or OpenSMILE features—optionally enriched with glottal source measures—provided the best unweighted recall (86.4%) for organic versus control classification. In their comparative analysis, ResNet-based encoders offered only incremental gains in multiclass scenarios [48,62]. Conversely, for high-dimensional inputs, convolutional and residual networks clearly outperform shallow models. Ma et al. showed that a TripleConvNet operating on mel-spectrograms and their temporal derivatives substantially outperformed single-input architectures for UVFP severity grading, reaching 91.5% accuracy compared to 84.5% for baseline CNNs [27]. Bur et al. reported ~92% sequence-level accuracy for benign versus malignant lesion classification using a ResNet-50 backbone on flexible laryngoscopic images [62]. Self-supervised encoders are particularly advantageous when labeled data are scarce, transferring representations from large speech corpora to pathology detection via simple downstream classifiers [23]. Clinically, this translates into a practical trade-off: feature-based shallow models suit binary screening and broad category recognition [39,40,48], whereas deep convolutional and self-supervised pipelines are justified for image-based diagnosis and fine-grained multi-pathology classification at the cost of reduced transparency [27,62,63]. Multimodal architectures integrating stroboscopic images, voice recordings and clinical variables show promise—Surapaneni et al. reported 76.9% accuracy on a held-out set versus 61.5% (video-only) and 65.4% (audio-only)—yet accuracy dropped to 45% on external validation, highlighting persistent domain-shift limitations [64].

#### 3.2.4. Validation Strategies: Internal Versus External

The distinction between internal validation, typically based on k-fold cross-validation within a single dataset, and external validation is crucial for assessing clinical generalizability of AI systems. External validation evaluates models on completely independent cohorts acquired at different centers or time periods. Most reviewed studies rely exclusively on internal validation procedures [13,39,45,65]. Internal cross-validation provides an initial estimate of model performance and helps to prevent overfitting to training data [25,65]. However, it does not guarantee robustness when models are deployed in new populations, with different recording devices, or in heterogeneous clinical environments. Several studies that explicitly implemented external validation have reported substantial performance degradation when models trained on one database are tested on another independent dataset. In some cases, accuracy and sensitivity decrease by about 10 to 30 percentage points under these conditions [16,64]. This consistent finding across multiple independent investigations underscores the critical importance of rigorous multicenter external validation. Such validation is required before any AI system can be considered ready for clinical deployment in real-world healthcare settings.

## 4. Diagnostic Performance and Methodological Biases

### 4.1. Synthesis of Reported Performance

Numerous studies report high diagnostic performance, with internal accuracies commonly exceeding 90% for AI-based detection of laryngeal disorders [22,24,44,66]. However, critical analysis shows that such performances are typically obtained under favorable experimental conditions. These conditions include historical databases dominated by sustained vowels, controlled recording environments, and predominantly internal validation, which are not fully representative of real-world clinical practice [6,7]. These headline accuracies must be interpreted with caution, as they likely overestimate expected performance in heterogeneous clinical settings [6,7,13,24]. On this basis, Table 2 provides a concise overview of the main methodological bias categories, their typical impact on reported performance and their implications for internal validation, external generalizability and clinical use. Across the included studies, selection and analysis biases commonly lead to optimism of about 8–15 percentage points in accuracy or unweighted average recall when performance is evaluated solely with internal cross-validation. Recent studies further clarify how architecture and modality influence these numbers. Classical ML models, primarily based on SVMs and Random Forests trained on handcrafted acoustic features, typically achieve internal accuracies ranging from 88% to 96% for binary healthy vs. pathological detection [25,48,65]. For instance, in the Malaysian Voice Pathology database, an online sequential learning machine reached 90% accuracy for normal vs. dysphonic classification, and 84–92% for more specific structural or malignant vs. benign distinctions [25]. In contrast, DL architectures, such as CNNs and RNNs, demonstrate superior flexibility, with accuracies frequently reaching 97% to 99% on standardized databases [3,26,30]. However, these DL models often obtain lower performance when the complexity of the task increases. Liu et al. used a one-dimensional CNN on stacked vowels from the SVD and reported a micro-averaged F1 score of 0.80 for three-class classification, compared with 0.77 for a baseline single-vowel model [67]. Beyond standard DL, recent self-supervised learning approaches (e.g., wav2vec 2.0) are beginning to bridge the gap between different recording protocols, maintaining robust performances, with unweighted average recall often exceeding 85% even in unstandardized acoustic conditions [23,28,29]. Hybrid ML-DL pipelines—where spectrogram features are extracted by a pretrained VGG-type network and classified by SVM—have achieved internal accuracies close to 98–99% on the SVD and maintained screening accuracies around 97% in small prospective clinical cohorts [7]. Studies demonstrate that sustained vowel audio systems tend to show the highest internal accuracies for binary classification, whereas image-based DL models generally perform better for detailed morphological lesion characterization, with reported sequence-level accuracies around 92% for benign vs. malignant lesions [65,67]. Multimodal architectures that combine voice recordings, electroglottographic signals and clinical variables report values in the 84–86% range, showing only marginal improvements over the strongest unimodal baselines [14,64].

### 4.2. Taxonomy of Methodological Biases

#### 4.2.1. Selection Bias

Strong dependency on a limited number of historical databases—primarily MEEI and SVD—acquired under specific clinical conditions introduces substantial selection bias [6,7,48]. The analysis of the literature suggests that this reliance on historical databases often leads to an overestimation of accuracy by approximately 8–15 percentage points compared to real-world clinical populations. Models trained exclusively on these corpora commonly show marked performance declines on independent cohorts, suggesting partial learning of database-specific artifacts rather than generalizable pathophysiological signatures [25,48,65]. Class imbalance further amplifies this bias by driving models to optimize for majority-class performance [6,39]. Most legacy databases comprise predominantly English- and German-speaking adults, while paediatric, geriatric, and non-Western populations remain largely absent from commonly used corpora [7,32,68]. Cross-linguistic and cross-database evaluations consistently report accuracy drops of 10–20 percentage points, reflecting phonetic, prosodic, and cultural mismatches in voice production [48,64,67].

#### 4.2.2. Measurement Bias

The overwhelming majority of reviewed studies rely on sustained vowel phonation recorded under controlled acoustic conditions, limiting ecological validity [6,7,31]. This measurement bias is critical, as models may rely on recording-related cues—such as background noise levels or signal-intensity differences between the groups—rather than true pathophysiology. Aichinger and Schoentgen illustrated this practically, as despite confirmed clinical diagnoses only approximately 70% of diplophonic participants produced detectable diplophonic segments during standardized recording tasks, suggesting that certain pathological symptoms are intermittent and context-dependent [19,49]. Additionally, historical databases sometimes employed different recording equipment for controls versus patients, introducing systematic technical differences that models may exploit as spurious classification cues [7,8,54].

#### 4.2.3. Analysis Bias

Reliance on internal cross-validation without external testing remains the principal analysis bias [13,33]. Models achieving high k-fold accuracy on a single dataset frequently show marked sensitivity drops when applied to data from new clinical centers [25,48,65]. This bias is characterized by a typical 12% performance drop when moving from internal cross-validation to external validation on unseen cohorts. Across the 88 included studies, only 7 studies performed both internal and external validation [12,16,26,54,64,65,69]. In these works, internal accuracies typically ranged from 80 to 98% whereas external accuracies fell between 45 and 77%, with individual drops reaching 20–30 percentage points in multimodal and multiclass settings. Many studies report only global accuracy without class-wise sensitivity, specificity, confidence intervals, or stratified demographic performance [34,48], allowing trivial majority-class classifiers to appear deceptively accurate in imbalanced datasets [70].

#### 4.2.4. Publication Bias

Reproducibility is rarely addressed explicitly in the vocal disorders AI literature [14,67]. This bias favors the reporting of near-perfect accuracies, leaving null or modest results underrepresented. As a result, this overestimates the true clinical readiness of these tools. Furthermore, a critical barrier is the lack of transparency; currently, fewer than 15% of the reviewed studies provide open access to their full model architecture or training code, severely restricting independent verification [13,14,33,34,48]. Selective submission and publication of high-accuracy results probably overrepresent optimistic performance scenarios and underestimate the true difficulty of robust clinical generalization [14,25,65,67].

### 4.3. Reproducibility and Inter-Center Variability

Only a small minority of studies provide sufficient open-source resources to enable full reimplementation, and available replication attempts consistently report substantial performance degradation [13,14,65]. Lee and Lee reproduced several published methods on a fixed SVD partition, obtaining unweighted average recall values 8–15 percentage points below original figures, suggesting dependence on undocumented pre-processing choices, hyperparameter tuning, or data partitioning strategies [48]. Cross-database evaluations confirm accuracy decreases of 10–20 percentage points when models trained on SVD or MEEI are applied to independent cohorts [25,48,65]. Low et al. further demonstrated that even within a single institution, subtle methodological differences in recording duration and mean intensity between patient and control groups generate spurious acoustic cues that artificially inflate internal cross-validation performance—an effect markedly attenuated once rigorous bias mitigation is applied [8]. Collectively, these findings suggest that reported high accuracies partly reflect overfitting to database-specific characteristics rather than genuine capacity to generalize across diverse clinical populations and recording conditions [25,48,65].

## 5. Current Clinical Recognition Levels

### 5.1. Three Level Frameworks for Clinical Recognition

**Figure 3:**
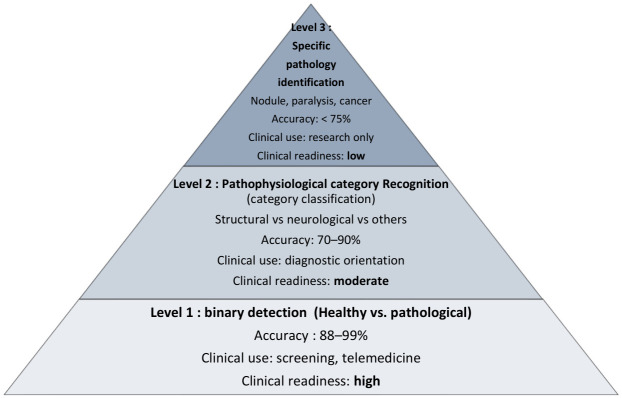
Three-level conceptual framework for AI-based clinical recognition of laryngeal disorders. In our corpus of voice-based AI studies targeting laryngeal pathology recognition, approximately 77.3% of studies primarily address level 1 binary detection of abnormal versus normal voice. Around 33% focus on level 2 classification into broader pathophysiological categories, and about 12.5% report level 3 performance for specific diagnostic entities. Because many studies inherently operate across more than one classification level, these percentages are intentionally overlapping distributions of research focus rather than mutually exclusive groups. These distributions correspond to 68 studies primarily addressing level 1, 29 studies reporting level 2 performance, and 11 studies reporting level 3 performance, within the set of 88 included studies.

To clarify the current state of AI-based laryngeal disorder detection, it is useful to distinguish three hierarchical recognition levels, each characterized by specific diagnostic tasks, typical performance ranges, and distinct forms of clinical utility. Across the corpus of studies included in this review, level 1 tasks consistently achieve markedly higher and more stable performance than level 2 and level 3 (Figure 3). By contrast, diagnostic performance at levels 2 and 3 exhibits wider variability and clear sensitivity to dataset composition and validation strategy.

#### 5.1.1. Level 1: Binary Detection (Healthy Versus Pathological)

The task is to distinguish voices exhibiting any pathological characteristics from completely healthy voices, irrespective of underlying etiology. Most studies on dysphonia detection belong to this category and typically rely on sustained vowels from historical databases under controlled conditions [5,6,30,71]. Reported performance at this level is consistently high. Accuracies commonly range between 88 and 99%, with sensitivities in the high 80s to high 90s and specificities generally between 85 and 95% when evaluated by internal cross-validation [6,7,24,44]. Studies with external validation report drops of 10–20 percentage points [25,65]. Level 1 systems are well-suited for telemedicine-based screening, pre-consultation triage, and use in resource-limited settings where specialist laryngoscopy is not immediately accessible. High sensitivity is paramount to minimize false negatives. However, specificity must be balanced to avoid overwhelming specialist services with false positives [5,67].

#### 5.1.2. Level 2: Pathophysiological Category Recognition

This level involves classification into broad pathophysiological families (three to five categories). These include structural mass lesions (nodules, polyps, cysts), incomplete glottic closure conditions (paralysis, paresis), neuromuscular disorders (spasmodic dysphonia, essential tremor), inflammatory conditions, and neoplastic lesions [47,72,73]. Reported accuracies at this level range from 70 to 90%, depending on the specific category combinations and datasets used [6,7,42]. Performance is highly variable depending on the category pairs being compared. Distinguishing structural masses from paralyzes may achieve accuracies of approximately 85–90%, whereas separating neuromuscular disorders from hyperfunctional patterns often falls to around 70–75% [42]. Level 2 recognition provides diagnostic orientation that can guide the selection of an appropriate imaging modality, referral pathway or specialist consultation type. However, acoustic overlap between pathophysiologically distinct conditions limits its reliability, such that disorders within the same category often still require morphological confirmation by laryngoscopy or imaging [19].

#### 5.1.3. Level 3: Specific Pathology Identification

This level aims at the precise identification of the underlying disorder, such as UVFP, Reinke’s oedema or laryngeal carcinoma. Only a minority of studies explicitly evaluate level 3 performance. Where reported, accuracies rarely exceed 75% and show substantial variability across the disorders [25,42]. Few studies describe accuracies above 80% for individual entities and such results typically reflect internal validation on small cohorts rather than robust external testing [25,72]. The fundamental acoustic overlap between pathophysiologically distinct conditions imposes an intrinsic performance ceiling that cannot be overcome by algorithmic sophistication alone [6,7,42]. Consequently, level 3 performance remains insufficient for autonomous diagnosis and must be integrated with laryngoscopy, imaging and histopathology for definitive diagnosis.

### 5.2. Recognition Level and Clinical Positioning

This three-level framework clarifies that current AI systems show robust and relatively consistent performance at level 1 [65], with moderate and highly variable performance at level 2 [70,74]. In contrast, they demonstrate clearly insufficient performance for autonomous use at level 3 [2]. This gradient is mirrored in individual multi-level studies. Works such as Lee and Lee, Morikawa et al., Naranjo et al., and the multimodal cohort reported by Surapaneni et al. all show high internal accuracies for level 1 and more modest and unstable results for level 2 [22,41,48,64]. When class granularity increases and external cohorts are considered, level 3 performance becomes clearly inadequate in terms of robustness. High reported accuracies primarily reflect methodological potential demonstrated under optimized conditions for binary abnormality detection. They do not reflect a present capacity to deliver specific otolaryngological diagnoses in heterogeneous clinical environments. Current dysphonia guidelines consider laryngoscopic visualization and multidimensional voice assessment as the reference standard [20,21]. In line with these recommendations, AI-based systems should be integrated as adjunctive tools that support, rather than replace, guideline-driven diagnostic pathways. Appropriate clinical positioning is to consider AI as a decision-support tool for triage screening and longitudinal monitoring at level 1 and selected level 2 tasks. It should be used as an adjunct rather than replacement for specialist evaluation and reference diagnostic methods [19,25,42].

## 6. Gaps and Perspectives

### 6.1. Underrepresented Disorders and Populations

Synthesis of the reviewed literature reveals three broad categories of research gaps that significantly limit clinical translation of current AI systems. First, several clinically important disorders remain substantially underrepresented in existing databases, e.g., spasmodic dysphonia, laryngopharyngeal reflux (LPR) disease, sulcus vocalis [9,39,51]. Second, most large databases primarily include adult speakers from limited linguistic and cultural backgrounds, predominantly German, English, and East Asian. Pediatric voices, geriatric voices, and speakers of under-resourced languages are underrepresented in the literature [32,48,68,75]. Third, only a handful of published works explicitly focus on clinically complex scenarios. These include patients with multiple concurrent disorders, post-surgical substitute voices following cordectomy or laryngectomy, and longitudinal monitoring of pathology evolution and treatment response over extended time periods [27,41,50,74,76].

### 6.2. Methodological Standardization Needs

At the methodological level, outcome measures and evaluation metrics used across published studies are markedly heterogeneous, complicating direct comparison and synthesis of findings. Many studies report only global accuracy metrics without providing detailed class-wise sensitivity, specificity, positive or negative predictive value, or confidence intervals stratified by pathology type, by severity, or demographic factors [25,34,48]. Risk-of-bias assessment tools are rarely used in AI literature [33,34]. Furthermore, transparency remains a major concern, as fewer than 15% of the reviewed studies share their source code, hindering reproducibility. Most studies rely on retrospective single-center data. There is a limited use of prospective study designs, pre-specified analysis plans or adherence to standardized reporting guidelines such as TRIPOD for prediction models or STARD for diagnostic accuracy [13,14]. Future work should adopt evaluation metrics that are carefully aligned with specific intended clinical use cases and incorporate structured bias assessment frameworks from the outset in a prospective design [14,34,48].

## 7. Recommendations for Future Research and Clinical Deployment

### 7.1. Research Priorities

Future research priorities lie not in incremental accuracy optimization within historical databases, but in methodologically robust protocols addressing fundamental generalizability gaps. Prospective multicenter databases should cover diverse disorders, languages, vocal tasks and recording conditions, reflecting real-world clinical heterogeneity [32,49,52,59,68]. This is crucial since 82% of current literature relies exclusively on sustained vowels, which may artificially inflate accuracy. External validation on independent cohorts from different institutions and time periods should become a mandatory publication standard [14,25,48,54,65], especially as performance often drops by 12 to 20 percentage points when tested on external data. Performance metrics must be stratified by pathology type, severity, demographic factors and recording conditions, and matched to the intended clinical use case. Screening applications require high sensitivity and negative predictive value to minimize missed diagnoses [34,48,65], whereas longitudinal monitoring demands measurement stability, calibration across repeated assessments, and explicit quantification of within-patient variability—with clinically meaningful change defined as exceeding typical short-term fluctuation [8,13,14,76,77,78]. Multi-class diagnostic settings require unweighted average recall and per-class sensitivity and specificity rather than global accuracy, which is misleading under class imbalance [34,48]. Research should further develop self-supervised representation learning to improve generalization in data-sparse regimes, alongside multimodal fusion strategies combining acoustic signals with laryngeal imaging and clinical metadata, while preserving computational tractability for real-time deployment [23,24,26,57].

### 7.2. Clinical Deployment and Integration into Practice

AI-based voice disorder systems should be positioned as complementary decision-support tools augmenting, rather than replacing, perceptual evaluation and laryngeal imaging. In telemedicine and primary care settings, level 1 binary detection models can support pre-consultation triage where specialist access is limited [39,40]. Clinical usefulness depends on external validation demonstrating sufficiently high sensitivity to minimize false negatives, with acceptable specificity to avoid overwhelming referral pathways [44,49,66]. At most, level 2 stratification may be supported, provided multicenter validation confirms adequate performance on consumer-grade recordings in realistic acoustic environments [22,42,46,50,59,66]. For patients under specialist care, level 1 and selected level 2 systems can assist longitudinal monitoring of treatment response by objectively quantifying temporal trends in acoustic parameters including percent jitter, shimmer, HNR, cepstral measures and ambulatory vocal load indices [43,57,74,76,78]. Accelerometer-based ambulatory monitoring in hyperfunctional disorders further demonstrates capacity to capture changes in vocal load and symptom severity over time [78]. In specialist practice, advanced level 2 and level 3 models may help prioritize cases at higher risk of malignancy or progressive neuromuscular impairment, prompting timely laryngeal imaging, biopsy or neurological referral [10,11,12,27]. Multimodal architectures combining stroboscopic video with acoustic and clinical features show promising internal performance for distinguishing benign from suspicious lesions [18,62,64], yet substantial degradation on external datasets confirms their current role as decision-support rather than autonomous diagnostic tools. Final diagnostic responsibility must remain with the treating clinician, with AI outputs interpreted alongside perceptual evaluation, laryngeal imaging, patient history and clinical context [34,54,79]. Prospective trials evaluating AI-augmented workflows against standard care—assessing diagnostic accuracy, time to diagnosis, resource utilization and patient-reported outcomes—are urgently needed [61,65,67].

### 7.3. Regulatory and Ethical Considerations

AI-based voice disorder systems increasingly fall under medical device regulations requiring structured evidence on training data quality, validation methodology and clinical benefit before routine integration [80,81,82]. In Europe, AI diagnostic software is regulated under the Medical Device Regulation framework, with most tools classified as Class IIa or IIb, requiring notified body assessment, clinical evaluation and post-market surveillance [83,84,85]. In the U.S., most AI/ML Software as a Medical Device is cleared as Class II via the 510(k) or De Novo pathways, with Food and Drug Administration communications emphasizing lifecycle management to address performance changes over time [82,86]. Although sharing broadly similar objectives, these frameworks differ in architecture, evidence requirements and regulatory timelines, with practical implications for international validation program design. Across jurisdictions, expert reviews converge on good development practices: clearly specifying intended clinical use and target population, prospectively designing external validation strategies, and maintaining traceable documentation of data, model architecture and update procedures [80,82,87]. Clinicians retain a duty of independent judgment over AI outputs, and governance frameworks must clarify responsibility allocation when AI-supported decisions contribute to diagnostic errors [80,87,88]. Key ethical considerations include algorithmic bias from demographic and linguistic under-representation, which may produce disparate performance across age, gender, ethnicity and language groups, raising equitable access concerns [25,32,58,65,68]. Informed consent must distinguish research from clinical use, and privacy frameworks must address the biometric sensitivity of vocal data [63,68]. Transparency regarding model limitations and failure modes is essential for justified clinician trust and appropriate deployment as decision-support rather than autonomous diagnostic tools [13,27,34].

## 8. Conclusions

AI-based systems for laryngeal pathology recognition achieve high diagnostic performance under controlled conditions, yet results are frequently obtained on historical databases with limited pathology spectra and internal validation, restricting generalizability. Our analysis of 88 studies highlights that 82% focus on static phonation and fewer than 15% provide an open-source code, contributing to a 12–20% performance gap in external testing. Acoustic overlap between pathophysiological entities, selection biases and absent external validation continue to limit clinical reliability. These systems cannot yet replace endoscopic assessment for specific diagnosis. Their optimal roles are as decision-support tools: level 1 systems for triage and telemedicine screening prioritizing sensitivity; longitudinal monitoring tools tracking within-subject acoustic changes to document treatment response; and advanced level 2–3 models assisting malignancy risk stratification and referral guidance. Final diagnostic decisions must remain grounded in laryngoscopy, imaging and histopathology.

## Figures and Tables

**Table 1:** Summary of voice databases used for AI-based laryngeal pathology recognition.

Base	N Healthy	N Pathological	Vocal Tasks	Recording Conditions	Language	Documented Biases
SVD	687	1356	Vowels /a/, /i/, /u/, at normal, high and low speech in the German sentence “Guten Morgen, wie geht es Ihnen?”	Phonetic lab recordings with studio microphone and electroglottograph, 16-bit, 50 kHz sampling, controlled acoustic environment	German	Highly unbalanced distribution; dominance of sustained vowels and read speech rather than spontaneous speech, limiting ecological validity [48,50,52]
MEEI	53	1319	Sustained phonation /a/ Reading of the first sentence of the Rainbow Passage.	Clinical recordings with Kay Elemetrics (Kay Elemetrics Corp., Boston, MA, USA); high-quality microphone, originally sampled up to 50 kHz	English	Class imbalance with far fewer healthy than pathological samples; tasks restricted to sustained vowels plus one standard sentence [6,7]
AVPD	188	178	Sustained vowels. Continuous speech. Isolated words.	Recorded with the Computerized Speech Lab (CSL 4500; KayPENTAX, Montvale, NJ, USA) using a studio microphone in a controlled clinical environment with a standardized protocol	Arabic	Mono-ethnic; predominance of sustained vowels compared with spontaneous speech [6]
VOICED	58	150	Sustained vowel /a/.	Recorded through a smartphone in a quiet room, 20 cm mouth-microphone distance, 8 kHz	Italian	Adult Italian speakers only; pathology spectrum limited to three main dysphonia groups [5]
FEMH	0	2106	Continuous speech: seven designed sentences per subject. Sustained vowel /a/.	Clinical recording environment, standard microphone, 16 kHz	Mandarin	No healthy control speakers; only four diagnostic groups; single-center mandarin cohort [12]

Abbreviations: SVD, Saarbruecken Voice Database; MEEI, Massachusetts eye and ear infirmary Database; AVPD, Arabic Voice Pathology Database; VOICED, Vox4Health m-health clinical study cohort (University of Naples Federico II); FEMH, Far Eastern Memorial Hospital voice disorder database.

**Table 2:** Major sources of bias affecting AI-based voice quality detection and their impact on internal validation, external generalizability and clinical reliability.

Key References	Bias Type	Typical Magnitude of Effect	Internal Validation	External Validation	Clinical Impact
[25,48,65,67]	Selection bias	Overestimation of accuracy by approximately 8–15 percentage points; Reduced detection of minority classes and rare disorders	Very high level 1 (binary) classification accuracy (88–99%) on sustained vowel datasets with balanced or curated samples	Typical decrease of 10–20 percentage points in accuracy and sensitivity when applied to independent or more diverse cohorts	Unequal diagnostic performance across age groups, languages and pathology subtypes, potentially leading to underdiagnosis in underrepresented populations
[8,19,31,42]	Measurement bias	Inflation of area under the curve and accuracy due to non-physiological cues such as recording conditions, signal intensity or duration rather than pathology-related features	Stable and homogeneous recordings (e.g., sustained vowel /a/, clinical-grade microphone) yielding optimistic and highly reproductible performance estimates	Significant performance degradation in noisy, ambulatory or telemedicine environments, reflecting reduced robustness to real-world variability	Models may rely on recording-related characteristics rather than pathophysiological features, increasing the risk of false negatives in real-world clinical settings
[13,25,48,65]	Analysis bias	Optimism of approximately 8–15 percentage points in accuracy or unweighted average recall compared to standardized or external validation procedures	k-fold cross-validation conducted on a single dataset frequently yields high accuracy, particularly in the presence of class imbalance or data homogeneity	Mean accuracy decreases of approximately 12 percentage points across studies performing both internal and external validation	Overestimation of true diagnostic performance, particularly for more complex classification levels (e.g., pathophysiological subtypes), with systematic under-detection of rare or subtle disorders
[13,14,34,48]	Publication and reproducibility bias	Reported accuracy and unweighted average recall often exceed independently reproduced performance by about 8–15 percentage points	Striking internal results obtained on single datasets are more likely to be submitted and published, while negative or modest findings remain underreported	Limited availability of code, pre-trained models or detailed methodological documentation; Independent re-implementations frequently report lower performance and incomplete reproducibility	Risk of premature clinical adoption of insufficiently validated tools, leading to overestimation of real-world reliability and potential patient safety concerns
[5,19,23,63,64]	Ecological validity bias	Large but difficult to quantify discrepancy between laboratory performance and real-world clinical effectiveness	Most models are trained and evaluated on sustained vowels or short read sentences recorded under controlled acoustic conditions, resulting in high level 1 classification accuracy	Reduced accuracy and increased variability observed when models are applied to continuous speech, spontaneous communication or smartphone recordings	Tools optimized for standardized speech tasks may fail to detect intermittent or context-dependent symptoms, increasing the risk of false reassurance and underdiagnosis in routine clinical practice

This table summarizes the main categories of methodological bias identified in the AI-based voice-quality literature, illustrating their typical magnitude, their effect on internal and external validation, and their potential clinical consequences. The estimates are indicative of ranges synthesized from multiple studies rather than pooled quantitative effect sizes and specific examples are detailed in the main text.

## Data Availability

No new data were created or analyzed in this study. Data sharing is not applicable to this article.

## Abbreviations

The following abbreviations are used in this manuscript:

AI	Artificial Intelligence
ML	Machine Learning
DL	Deep Learning
VQ	Voice Quality
F0	Fundamental frequency
HNR	Harmonic-to-noise ratio
UVFP	Unilateral Vocal Fold Paralysis
MEEI	Massachusetts Eye and Ear Infirmary Database
SVD	Saarbrücken Voice Database
AVPD	Arabic Voice Pathology Database
VOICED	Vox4Health m-health clinical study cohort
FEMH	Far Eastern Memorial Hospital voice disorder database
CNN	Convolutional Neural Network
LSTM	Long Short-term Memory
MFCC	Mel-Frequency Cepstral Coefficient
U.S.	United States
